# Our New York Letter

**Published:** 1890-10

**Authors:** Wm. L. Russell

**Affiliations:** New York


### Correspondence.

### OUR NEW YORK LETTER.

NEW YORK, September 15, 1890.

### CREOSOTE IN CONSUMPTION.

The administration of creosote for pulmonary phthisis has become the principal feature in the medicinal treatment of that fatal disease. Dr. Beverly Robinson, of this city, who may be regarded as the apostle of this plan of treatment in this country, has done much to lead to its general adoption. Speaking on the subject at the last meeting of the Clinical Society, he stated that he believed the remedy to be the best in our possession, and so valuable that he hoped the observations which had been made would be regarded as conclusive, and would make it almost compulsory to administer creosote in every case of phthisis. His custom was to give from half a minim to a minim five or six times in twenty-four hours. It was important to have a pure creosote, well diluted, and perfectly dissolved, in order to guard against disturbance of the stomach. A small dose should be given for a long time; then a very gradual increase should be made until the point of tolerance was reached, when the dose should be continued uninterruptedly for several months. Given in this way he had seen great good result. The sputa diminished and disappeared, nutrition improved, strength increased, and the cough was arrested. In two cases he had seen the bacilli disappear from the sputa. In several cases he had seen recovery, and this was more than he had ever seen in dispensary or hospital patients treated by cod liver oil and hypophosphites. He belived that the combined administration by stomach and by inhalation would prove most satisfactory when the patient would tolerate it.

At the same meeting Dr. W. H. Flint gave a short historical review of the creosote treatment, and mentioned a number of formulæ for administering it. Among them was that used by Dr. Robinson for inhalation. This consisted of one drachm of creosote in half an ounce of alcohol. Ten to twenty drops of this were to be placed on the sponge of an inhaler three or four times in the twenty-four hours. The inhaler should then be worn for fifteen or twenty minutes every three hours, at first, and for a longer period as the patient grew accustomed to its use. For internal administration the following was said to prove satisfactory :

℞. Creosoti (beechwood),............... m. vi.
Glycerinæ.......................... ℥i.
Spt. frumenti...................... ℥ii.
M. S.

A teaspoonful every three hours diluted with two parts of water. Another formula was one used at Roosevelt Hospital by Dr. Newcomb, and consisted of an emulsion made as follows:

℞. Creosoti..........................

Tinct. Capsici..................... *aa* ʒii-ʒiii.

Mucil. Acaciæ...................... ℥ss.

Aq................................. *ad.* ℥iv.

M. S.

A teaspoonful well diluted with water after meals.

Dr. Flint had found that an emulsion of cod liver oil was the most satisfactory vehicle in which to administer the remedy. This could best be given shaken up with milk in a punch shaker. The creosote imparted a rather unpleasant taste to the milk, but this was usually soon tolerated, and a larger dose could be given in this than in any other way. Ten or fifteen minims daily was the maximum dose, which most patients would tolerate. When the stomach or palate rebelled against even a moderate dose, the emulsion could be readily given by rectum. Intra-pulmonary injectives had not given general satisfaction, and were of doubtful value.

BROMOFORM.

Bromoform is one of the newer remedies which promises to be extremely useful. It has been tested by a number of observers, and has given general satisfaction. Its special value is in whooping cough, and all practitioners will be glad to know that in it has been found an apparantly safe agent for the amelioration and cure of this disease. It is a colorless liquid, produced by the action of bromine on alcohol in the presence of an alkali. It is an anæsthetic, and has been used as such. The most marked feature of its action being pronounced cyanosis. Dr. Stepp, of Nuremberg, first used it in whooping cough, and later Prof. Senator, of Berlin, tested it and gave it his endorsement. It was stated by these observers that ordinary cases were, as a rule, benefited as early as the second day, and severe cases as early as the third or fourth day. Vomiting disappeared during the first week, and any complicating bronchitis improved. The appetite also improved, and a cure resulted in from two to four weeks. Relapses occurred if the treatment were not persisted in. Sleepiness was noticed in a few cases. Poisonous symptoms caused by the drug were markedly contracted pupils, small, compressible pulse, pale countenance and insensitive corneæ; auscultation showed long, deep inspiration, with very short and feeble expirations, and the heart sounds were scarcely perceptible.

Dr. Louis Fischer, of this city, from whose article in a recent number of the *Medical Record* the above has been taken, has reported the histories of sixteen cases in which he had used the remedy. Most of them were from among poor dispensary patients who live in very unhygienic surroundings. Some, however, were from the better classes, and all irrespective of surroundings were similarily benefited. He required the parents to note the exact number of paroxysms during a given number of hours before and after treatment was begun. The result showed marked benefit from the use of bromoform. In most cases the cough ceased entirely after nine days of treatment, and was reduced to one paroxysm daily by the fifth day. In some cases eighteen days were required to effect a cure, and in a few, even a longer period. The dose given was two drops three times daily to children up to one year old. Children from two to four years old were given three or four drops, and those older, up to eight years, four to six drops three times daily. On the third or fourth day it was his custom to increase the dose by one drop. The medicine should be given after food in a small teaspoonful of water. It sank to the bottom of the teaspoon, and care was necessary to insure its administration. It was seldom objected to by the patient, as the taste was quite pleasant.

SALOL.

Still another comparatively new remedy is salol. It was first used in 1886. It is a coarse, white powder, with an aromatic odor, and a faint, rather agreeable, taste. It is insoluble in water, but soluble in alcohol and ether. It is decomposed by sodium-bicarbonate, which should, therefore, not be administered with it. It can be easily emulsified. It is insoluble in the stomach juices, but dissolves readily in the alkaline fluid, of the intestines. There, it is decomposed into its primary constituents, salicylic acid and the phenol group, and it has thus come to be used in the summer diarrhœa of children. The carbolic acid passes off in the urine, and for this reason the remedy has been used in pyelitis and cystitis. It has also been used in rheumatic affections, but its special value is in the treatment of acute pharyngitis and tonsillitis.

Dr. Johnathan Wright, of Brooklyn has used it in a number of these cases, and has found that it will relieve the pain and discomfort in from twenty-four to forty-eight hours. It is of least value in quinsy, and it fails occasionally in all kinds of cases. Besides the relief which it affords, it reduces the temperature. The dose required is from one to two drachms daily, a drachm and a half being the usual quantity. It can be given in powders or emulsion in ten grain doses every two hours.

NOCTURNAL ENURESIS.

An ingenious and simple method of treatment for a very troublesome disorder was suggested at the International Medical Congress by Dr. Van Trenton, of La Hayne. He stated that the nocturnal enuresis of children was due to insufficiency of the sphincter vesicæ, which allowed the urine to flow into the upper portion of the urethra, from which it was then expelled by reflex action of the detrusor urinæ. The fact that the child wetted the bed two hours or so after going to sleep, proved that it was not due to distention of the bladder. His treatment then consisted in preventing the urine from running into the urethra by raising the foot of the bed. He had cured fourteen children in this way, taking the additional precaution of having them empty the bladder just before retiring, and of giving them no liquid at this time.

### ERYSIPELAS.

The latest treatment of erysipelas is by linear scarification and friction, with a solution of mercury bichloride 1-1,000. A number of cases have been treated here in this way, and quite successfully. The mode of procedure is to scarify around the limb a short distance above the upper border of the disease, or around the seat of disease if it be not on a limb, and to rub the solution into the scarified region. If this is done thoroughly the disease seldom crosses the barrier. In one case in which it did cross, a second circle was formed which proved successful. The scarification can be done very quickly with an ordinary scalpel, but a multiple scarifier or "harrow" has been devised by Prof. Seibert, which need only be drawn around the limb. The objection to this treatment is, of course, its painfulness, but when the disease cannot be limited by ordinary methods, this is well worthy of a trial.

WM. L. RUSSELL.

